# Evaluating the Application of the Mental Model Mapping Tool (M-Tool)

**DOI:** 10.3389/fpsyg.2021.761882

**Published:** 2021-12-14

**Authors:** Karlijn L. van den Broek, Joseph Luomba, Jan van den Broek, Helen Fischer

**Affiliations:** ^1^Research Centre for Environmental Economics, University of Heidelberg, Heidelberg, Germany; ^2^Copernicus Institute of Sustainable Development, Utrecht University, Utrecht, Netherlands; ^3^Tanzanian Fisheries Research Institute, Mwanza, Tanzania; ^4^Department of Theoretical Epidemiology, Utrecht University, Utrecht, Netherlands; ^5^Max Planck Institute for Human Development, Berlin, Germany

**Keywords:** mental models, cognitive maps, methods, systems thinking, perceptions, participatory modeling, M-Tool

## Abstract

Mental models influence how individuals think and act in relation to their external environment and have been identified as leverage points to address sustainability challenges. Given the importance of mental models, a new tool to assess mental models has been developed: the Mental Model Mapping Tool (M-Tool). M-Tool was designed to have a standardized format and to be user-friendly for low literacy populations, using pictograms and audio instructions. In this paper, we evaluate M-Tool’s application in two studies with Tanzanian fishers. In Study 1, we investigated M-tool’s convergent validity compared to standard interviewing methods (*n* = 30). Study 2 investigated M-Tool’s construct validity by relating mental model complexity to participants’ education level (*n* = 185), a relationship that has been well established. The findings show that (1) mental models produced with M-Tool are more complex than mental models obtained through interviewing techniques; (2) model composition is similar across the two methods; and (3) participants with higher levels of education tend to produce more complex mental models with M-Tool than participants with lower levels of education, in line with previous research. These findings suggest that M-Tool can successfully capture mental models among diverse participants. This tool offers researchers and practitioners an instrument to map and compare perceptions of (conservation) challenges across groups.

## Introduction

Mental models are internal representations of the external world consisting of causal beliefs that help individuals deduce what will happen in a particular situation (Craik, 1943; Johnson-Laird, 1989; Bostrom, 2017). These cognitive structures reflect an individual’s implicit or explicit assumptions about how things function, which can be inaccurate or incomplete as they are a simplified representation of reality (Vennix, 1999; Morgan et al., 2002; Johnson-Laird, 2010). Mental models are developed based on experience, culture, values, and beliefs (Biggs et al., 2011; Bender, 2020), are malleable, and likely to change (Norman, 1983).

Mental models are often activated automatically (Fiske, 2014) and consequently influence how individuals filter, process, and store information (Genter and Stevens, 1983; Johnson-Laird, 1983; Kempton, 1986; Nersessian, 1992). Therefore, mental models form the foundation for explaining events, reasoning, and predicting future developments (Jones et al., 2014). Furthermore, they guide people’s attitudes, judgments, decision-making processes, and actions (Morgan et al., 2002; Biggs et al., 2011; Güss and Robinson, 2014; Goldberg et al., 2020). For example, mental models have been found to correspond to policy preferences: in a study on climate change mental models, the perceived cause of climate change predicted the preferred mitigation policy (Bostrom et al., 1994, 2012).

The concept of mental models has been identified as a leverage point within psychology research for addressing sustainability challenges such as climate change (Goldberg et al., 2020). Mental models can be targeted to (1) foster system thinking, which can encourage pro-environmental attitudes and actions (Goldberg et al., 2020), (2) identify consistencies and disparities in perceptions and beliefs between individuals (Wood et al., 2012), or (3) identify misperceptions that can be addressed in risk communication (Morgan et al., 2002).

Since mental models are internal cognitive constructs, they are not readily available to directly measure or analyze (Eden et al., 1992; Jones et al., 2011). Hence, a range of cognitive mapping methods exists to elicit mental models (Jones et al., 2011; Moon et al., 2019). Diagram drawing methods are commonly used to obtain a representation of a mental model as they make implicit assumptions explicit in a visual way (Abel et al., 1998). Specifically, influence diagrams are directed graphs that show the structure of the mental model, including the relevant concepts and the directional relations between those concepts (Johnson-Laird, 1983; Wood and Linkov, 2017). The relations between concepts are often depicted by arrows that indicate the perceived strengths and direction of the influence and whether the associations are positive or negative (Cox et al., 2003). Influence diagrams have been used to understand mental models in the areas of sustainability (Lowe and Lorenzoni, 2007; Tschakert and Sagoe, 2009; Bardenhagen et al., 2020), environmental hazards (Atman et al., 1994; Linkov et al., 2009; Bostrom et al., 2016), and health (Fischhoff et al., 2006) to represent perceptions that influence decision-making.

Influence diagrams can be constructed by the researcher, based on interviews with participants or surveys (indirect elicitation), or the diagrams are drawn directly by the participants themselves (direct elicitation; Jones et al., 2011). A method that uses a mix of these approaches is the risk communication mental model approach (Morgan et al., 2002). With this method, experts construct an influence diagram, which provides the reference point to analyze lay mental models assessed through semi-structured interviews. The lay mental models are compared against the expert influence diagram to identify common (mis)perceptions in the mental models. The prevalence of these (mis)perceptions is then verified with a survey with a larger sample and can be addressed through risk communication (Bruine de Bruin and Bostrom, 2013). Another common mental model elicitation method is the fuzzy cognitive mapping approach with which participants directly create the influence diagrams (Özesmi and Özesmi, 2004). This method is often used to elicit expert knowledge and forms the basis for scenario analysis to determine how the system might react under a range of possible changes. Direct methods of mental model elicitation have the benefit that participants can verify the resulting influence diagram (Jones et al., 2014) and facilitate the comparison of experts with lay knowledge (Gray et al., 2013).

### A Need for Standardization in Mental Model Elicitation

Comparing mental models across groups can reveal important differences in beliefs and knowledge and can help identify commonalities and disagreements that can inform risk communication or conservation management (Wood et al., 2012; van den Broek, 2018). When methods are not standardized, comparison of mental models across groups may be challenging. Standardized methods streamline the assessment procedure for all participants to eliminate random noise that is confounding with the methods (Timmermans and Epstein, 2010).

In many mental model studies, participants generate their own concepts while constructing their cognitive map (Henly-Shepard et al., 2015). Without a fixed set of concepts, participants’ freely associated concepts need to be homogenized to resolve variations in language (Mourhir, 2020). This is often done qualitatively by classifying the concepts into overarching categories and using these categories as nodes in mental model network analysis (Özesmi and Özesmi, 2004; Olazabal et al., 2018). Researchers may also need to judge the redundancy of concepts and eliminate those that are perceived to be synonyms or align concepts with opposite directions of the same concept (Vasslides and Jensen, 2016). Such a process is resource-intensive (Mourhir, 2020) and sensitive to the researcher’s influence. Hence, some studies employ a two-step approach in which the mental model concepts are first generated through interviews or the literature reviews. Next, a fixed set of concepts is provided to participants to construct their cognitive map. This approach ensures that cognitive maps consist of the same set of concepts across participants and researchers do not have to homogenize the concepts (e.g., Gray et al., 2015; Aminpour et al., 2020).

Another important consideration for standardization in mental model research is the risk of capturing random noise in mental models, or even systematic error stemming from unstandardized instructions, facilitation, and other unwanted influences of the researcher or methods on the data. For example, interviewers can influence the interviewee’s responses simply through differences between the interviewer and interviewee in experience, nationality, or race, particularly for sensitive issues (Loureiro and Lotade, 2005; Olson and Peytchev, 2007; Samples et al., 2014). Therefore, mental model elicitation methods could benefit from reducing the researcher’s involvement in the elicitation process and instead provide participants with a uniform and computerized format with standardized instructions.

Indeed, mental model researchers have called for further development of visual mental model elicitation methods to ensure robust and reliable elicitation and allow systematic cross-group comparisons (Rouwette and Vennix, 2006; Schaffernicht and Groesser, 2011; Wood et al., 2012). Mental model elicitation methods can be standardized by providing participants with a fixed set of concepts to create their mental model and by reducing the role of the researcher with a standardized format and instructions. Because standardized methods facilitate the process of aggregating individual mental models, such methods provide opportunities to systematically compare different stakeholder groups. However, to be able to compare different groups of stakeholders, the methods not only need to be standardized, but also inclusive.

### Assessing Diverse Views in Mental Model Research

Although human–environment interactions are culture-bound, non-WEIRD participants (Western, Educated, Industrialized, Rich, Democratic; Arnett, 2008) are underrepresented in (environmental) psychology research (Henrich et al., 2010; Rad et al., 2018; Tam and Milfont, 2020). In fact, WEIRD participants are not representative of the global population in terms of their visual perception, spatial reasoning, categorization, inferential induction, moral reasoning, and self-concepts (Henrich et al., 2010). Hence, homogenous samples may limit the generalizability of research (Henrich et al., 2010; Bryan et al., 2021) and may hamper mental model theory development and its applications (Agrawal, 1995; Huntington, 2000). For example, a recent study found that by aggregating mental models of diverse groups of stakeholders, the mental models more closely matched the scientific understanding of the system than the mental model of a single group of stakeholders (Aminpour et al., 2020). This effect may be due to heterogeneous groups increasing the likelihood of uncorrelated judgment errors where, consequently, pooled judgments average out errors between individuals, an effect referred to as the wisdom of the crowd. Hence, scholars have called for more diversity in environmental psychology research (Milfont and Schultz, 2016) and suggested that including non-WEIRD samples should merit higher interest of editors and reviewers (Rad et al., 2018).

However, the current mental model elicitation methods that are most suitable for rigorous comparison may not be sufficiently inclusive. One issue is that many methods require participants to read and/or write. Although illiteracy rates have been steadily declining in the past few decades, low literacy rates (the proportion of the population that has difficulty with reading and writing) are still prevalent across the world. Low literacy rates are not only prevalent in developing countries but also in countries such as the Netherlands, where 12% of the population between 18 and 65 years are classified as low literate (Stichting lezen en schrijven, 2019). Furthermore, research on children’s mental models may also benefit from, or require, methods that do not rely on the participants’ literacy.

Most mental model studies with low literacy participants have relied on (semi-structured) interviewing techniques or focus group methods with which a facilitator distils mental models from discussions (Tschakert and Sagoe, 2009; Halbrendt et al., 2014; Nyaki et al., 2014; Rivers et al., 2018; Mehryar et al., 2019). Such methods rely on the researcher’s interpretation of participants’ discussions to construct the mental model (Kearney and Kaplan, 1997). Furthermore, the interview and analysis process tends to be time-consuming, often resulting in small sample sizes and limitations to the generalizability of the findings (Kearney and Kaplan, 1997). Moreover, these methods tend to rely on the participant’s ability to verbally articulate complex connections within a system, which may be particularly challenging for populations with lower levels of formal education. Hence, mental model research with low literacy participants can particularly benefit from more standardized and inclusive methods.

Visual materials are effective communication approaches to convey instructions to low literacy populations, such as migrant workers, to overcome language barriers (Caffaro et al., 2020; Vigoroso et al., 2020). Hence, visual approaches to mental model elicitation that may be more suitable for low literacy populations include card or photograph sorting tasks or a free drawing exercise (Bostrom et al., 1992; Chisik, 2011). However, we are not aware of any standardized methods designed to be user-friendly for diverse participants that employ a pictogram-based approach in influence diagram drawing tasks. Therefore, we evaluate a recently introduced standardized pictogram-based mental model elicitation tool, M-Tool (van den Broek et al., 2021), and test its suitability for low-literacy populations. With this tool, participants create influence diagrams using a fixed set of concepts. Participants choose which concepts they want to include in their models and connect them with weighted, directional arrows. The set of concepts can be obtained through interviews or surveys with a small but reasonably representative sample or through a literature review.

The participant works through four parts in M-Tool: (1) an introduction video, (2) a practice task, (3) a description of the pictograms representing the concepts, and (4) the mental model mapping screen. The introduction video demonstrates the use of M-Tool by working through a practice task. On the next screen, participants replicate the practice task using the same pictograms and arrows. The third screen displays a video with a description of each pictogram. The final screen consists of the mental model mapping screen. This screen is accompanied with audio instructions that inform the participant to move relevant concepts to the middle of the screen, choose which arrow-width they want to use to connect the concepts, listen to the explanations of the pictograms again, and delete pictograms or arrows if they like to.

### Overview of the Present Research and Hypotheses

We tested the usability and validity of M-Tool in two studies with Tanzanian fishers at Lake Victoria. According to local stakeholders, the key challenge in this region is the declining Nile perch fish stock, but the cause for this trend remains unclear, and perceptions on this seem to differ widely (van den Broek, 2019; Klein et al., 2021). The importance and diverse views on the drivers of the Nile perch stock fluctuation make it a suitable topic for mental model elicitation. The current study was preceded by a co-development process with Lake Victoria stakeholders to develop the research agenda for an interdisciplinary research project on tipping points. We followed a 10-step co-development framework to guide this process, starting with establishing a framework for the project, followed by a stakeholder and problem analysis, which led to an iterative process of research concept development and stakeholder feedback (van den Broek et al., 2020). The Mara and Mwanza region in Tanzania were selected to represent both rural and urban riparian regions with various levels of education and literacy at Lake Victoria to provide a challenging test for the tool.

M-Tool’s validity was evaluated in two studies. Study 1 assessed M-Tool’s convergent validity by comparing M-Tool with an alternative elicitation technique appropriate for measuring mental models among populations with low literacy: semi-structured, face-to-face interviews (Findlater et al., 2018). With a within-subject design, this study applied both methods to each participant to investigate the benefits and costs of restricting participants to a more standardized approach. M-Tool mental models were expected to be more complex compared to interview mental models (Hypothesis 1), due to lower working memory demands of the visual diagramming task (Suwa and Tversky, 2002). The different elicitation techniques were expected to produce broadly similar results with respect to model composition (Hypothesis 2), meaning the concepts and connections between the concepts in the mental models.

With a larger sample in Study 2, M-Tool mental models were related to participants’ level of education to assess the construct validity of the tool. The positive association between education level and complex thinking has been established in the field of cognitive psychology (Jaques, 1986; Perkins and Grotzer, 2000) and confirmed in mental model research (Levy et al., 2018; Varela et al., 2020). Hence, if M-Tool produces valid mental models, education levels should also be related to M-Tool mental models. Therefore, higher levels of formal education were expected to be associated with more complex mental models (Hypothesis 3).

## Study 1

### Materials and Methods

The first study aimed to investigate the convergent validity of M-Tool (a direct elicitation method) compared to standard interviewing methods (an indirect elicitation method) with a within-subject design. Convergent validity demonstrates the extent to which different measure instruments designed to capture the same construct relate to each other (Cunningham et al., 2001). Convergent validity has been assessed previously to validate other mental model elicitation methods (Daniels et al., 1993).

Alternatively, M-Tool could have been compared to a paper-based diagramming task, in which participants construct their mental models with a set of cards displaying the pictograms. Comparing such a method with M-Tool is likely to result in perfect replications of the mental models across the two methods due to participants’ motivation to appear consistent, which may not be very informative of the usability of M-Tool. Instead, comparing M-Tool to interview methods provides a more stringent test. Specifically, similarity in the content of the mental models across the two methods would indicate that we adequately captured the most important drivers from the previous semi-structured interviews (van den Broek, 2019) and that participants can reproduce their mental models with M-Tool.

Data were collected in collaboration with the Tanzania Fisheries Research Institute (TAFIRI Mwanza). Two research assistants conducted the interviews and assisted participants with M-Tool. The research assistants were thoroughly trained prior to the interviews, to avoid introducing terminology or influencing the terms of the discussion (Arthur and Nazroo, 2003). Practice sessions were conducted to refine the interview procedure before field pre-testing with fishers.

#### Participants

The sampling strategy was developed to target Nile perch fishers with little technological experience and low levels of education. We employed a time-location sampling strategy, which first involves sampling locations where individuals of interest can be found, and then sampling those who are present at the sampled locations at the time of sampling (Karon and Wejnert, 2012). Three landing sites were randomly selected from a list of all landing sites with more than 40 fishing boats targeting Nile perch (to ensure a sufficiently large participant pool) in the Mara region in Tanzania. At each landing site, 10 fishers expected to meet the inclusion criteria (adult Nile perch fishers) were randomly selected from a list of all fishers registered at the landing site, or from all fishers available at the time of the survey. Landing sites were visited at times that most fishers were expected to be available. Participants were financially compensated for their time after completing the study.

Participants (*n* = 30) were experienced fishers (Years of fishing_mean_ = 13.97, Years of fishing_SD_ = 7.34), who functioned as crew members on the fishing boats (80%) or were boat-owners (20%), and were fishing with gillnets (60%) or longlines/hooks (40%), a ratio that is representative for the fishery (Msuku et al., 2011). Participants were all men, who had attained low levels of education (3.3% no education, 86.7% primary education, 10% secondary education), which also representative for the Tanzanian Lake Victoria fishery (Luomba et al., 2013), and included a wide age range (age_mean_ = 38.57, age_SD_ = 9.10). Considering participants’ low levels of education, and remote areas of the sampling locations, the sample strategy was successful.

#### Procedure

The participant information sheet was read out to participants, who provided informed consent orally. Next, the sessions started with a semi-structured interview, followed by the M-Tool task. Before concluding the session, participants filled in a short survey with the help of a research assistant. It was not possible to counterbalance the order of the interview and M-Tool, as the latter tool provided participants with the driver concepts for the mental models, which was likely to influence the concepts participants would raise during the interview. Instead, the order of the tasks helped to assess if participants could represent their views with M-Tool.

#### Interview

The research assistants, who were well acquainted with local communication styles and cultures, conducted semi-structured interviews in Kiswahili. The interview consisted of four questions that were to simulate the information obtained through M-Tool. First, the topic was introduced to participants (“*Have you seen any changes in the size of the Nile Perch stock?*”). Next, participants were asked for the drivers in their mental model (“*What are the causes of these changes in the Nile Perch stock?*”), and their relative influence to assess the strength of the connections in the model (“*Which of these causes are more important and which are less important?*”). Finally, participants were asked to connect the different drivers in their mental model (“*Do these drivers influence each other as well?*”). When participants were hesitant to elaborate, the research assistant used standard prompting phrases (e.g., “*Can you think of any more causes*…?”). Due to the limited number of questions, interviews were fairly short (Minutes_mean_ = 8.66, Minutes_SD_ = 3.29). Discussions were audio-recorded and transcribed verbatim in Kiswahili by the same research assistants who conducted the interview. A third research assistant translated the transcriptions to English, and these transcripts were checked and edited by the authors.

#### M-Tool

To tailor M-Tool to assess mental models of the drivers of the Nile perch stock fluctuations, a set of driver concepts needed to be developed (for a guide on how to set up the tool for any research project, see van den Broek et al., 2021). In a previous study, we have conducted semi-structured interviews with Lake Victoria stakeholders (*n* = 67) representing 26 different institutions on the shores of Lake Victoria in Kenya, Tanzania and Uganda (including seven governmental organizations, 10 NGO’s, three fish processing businesses, three research institutions, and three community groups; van den Broek, 2019). In these interviews, participants elaborated on the drivers contributing to the declining Nile perch stock. From this study, we selected the drivers discussed in three or more interviews, resulting in 15 concepts driving changes in Nile perch stock. Limiting the number of drivers ensured that we captured the key components of most fishers’ mental models. Including the additional 12 drivers may have helped some participants describe their mental models in more detail; however, in line with previous research (Özesmi and Özesmi, 2004), we reduced the number of concepts to the top 15 drivers because presenting 27 drivers may have overwhelmed participants.

A graphic designer created two sets of pictograms representing these 15 concepts, which were tested in a pilot study with 20 Tanzanian Lake Victoria fishers. The definitions of the concepts were read out, and participants chose the images that they thought best represented the definition. The most frequently chosen pictograms were included in M-Tool (see Supplementary Material A and B for the pictograms and definitions, respectively). We acknowledge that abstract concepts (including open access to the lake and awareness of sustainable fishing practices) were inevitably difficult to represent in pictograms. Therefore, care was taken to include clear audio descriptions that closely matched the way participants had described these concepts in the previous study and were similar in structure and length to avoid drawing unequal attention to pictograms.

**Figure 1:**
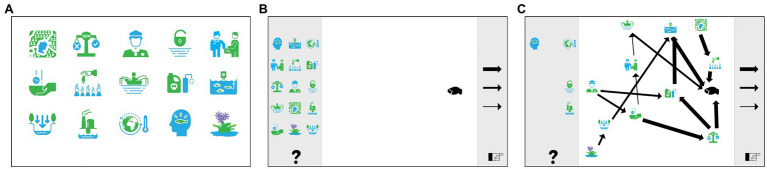
Screenshots of M-Tool: **(A)** presentation of the pictograms, **(B)** the mental model mapping screen, and **(C)** example of a participant’s cognitive map drawn in M-Tool.

In the current study, participants interacted with M-Tool on a tablet, using earplugs to listen to the instructions on the tablet. Participants worked through the practice task and listened to the audio descriptions of the pictograms (Figure 1A). Upon opening this mapping screen, audio instructions played automatically, first instructing participants to listen to the descriptions of each pictogram again (Figure 1B). Then for each concept, participants decided whether it influenced the Nile perch stock, and if so, moved it to the drawing board on the screen. Next, participants were told to organize the pictograms, connecting pictograms using the three arrows, which size correspond to the strength of the influence. When participants were unsure what to do, the research assistant reiterated the audio instructions by asking which concepts influenced each other and to connect the drivers that directly influenced the Nile perch to the Nile perch pictogram. An example of a cognitive map drawn in M-Tool can be seen in Figure 1C.

Participants were encouraged to walk the research assistant through their thought process and explain each connection made in the model. This procedure ensured that the connections reflected participants’ thought processes and let the research assistant verify they understood the task. Research assistants only demonstrated the interaction with the software during the practice task and did not interact with the software during the mapping of the mental model to avoid influencing participants’ mental models or leading the participant. Although a higher level of involvement of the research assistant may have made for a more naturalistic setting, the endeavor to standardize the instructions and procedures required structured and minimal involvement of the research assistant. Furthermore, the participant created their own diagram rather than having the research assistant interact with the tool for them, to avoid the need to interpret participants’ discussions. The active involvement of the participant in the creation of the model was expected to prompt more ownership of the model and more careful construction of the cognitive map, resulting in a more accurate reflection of their mental model. The mapping exercise took participants 23.33 min on average (SD = 8.7 min), which could be considered a short amount of time to elicit complex mental models compared to the 40–60 min reported in some mental model literature (Özesmi and Özesmi, 2004; Gray et al., 2012). However, such estimations tend to include the instructions and practice task, the generation of concepts by participants, and mapping mental models of the entire system, rather than focusing on the drivers only.

Before the data collection for Study 1 commenced, we conducted a pilot study with six participants at a landing site near the research center in Mwanza, to assess if any challenges would occur that needed to be addressed. This pilot showed that participants were slightly intimidated by the interaction with the tablet and did not tend to initiate the mental model task after the audio instructions were finished. Following this, the audio instructions were updated to be more structured and easier to comprehend. Furthermore, a script was written for the research assistants to guide participants in a way that was consistent with the audio instructions and across participants.

#### Survey

At the end of the session, participants filled in a short paper and pencil survey that collected demographic information (among other items that will not be included in the analysis for this study). Items assessed the participants’ age, gender, role in the fishery (boat owner or crewmember), number of years they had been fishing, used fishing gear (long-line/hooks or gillnets), and education level. Participants also reported on their experience with the interaction with M-Tool (“*How did you feel about using the tablet to represent your views about the Nile perch stock?*”). Research assistants read out the questions and response options to participants and filled in the participants’ responses.

#### Data Analysis

M-Tool was evaluated by analyzing (1) participants’ feedback to assess the user experience, (2) the differences in complexity between the interview mental models and M-Tool mental models, and (3) the agreement in the composition between the interview mental models and M-Tool mental models.

For each participant, the cognitive map was represented in edge lists, listing one row for each connection made for each participant, including the starting point, endpoint, and weight of the connection. This edge list was read into analysis software R using the M-Tool data analysis script (van Boxtel and van den Broek, 2021), to compute the complexity indicators. To compare M-Tool and interview mental model data, a similar data-structure was required; hence, the interview data were coded using the M-Tool framework. Despite the extensive training of the research assistants, the transcript revealed several suggestive questions posed by the research assistants in the interview (e.g., “*What brought about these changes? Are fishers more aware or forced to comply?*”). Hence, the transcripts were thoroughly inspected for suggestive questions, and upon agreement between two authors, were coded as such. Direct responses to these questions were omitted from further coding, and drivers that the interviewer had previously suggested were not coded in the remainder of the interview, exercising a conservative approach to save-guard the validity of the data. Statements that referred to drivers that influenced the Nile perch were coded using the 15 driver concepts included in M-Tool. Discussions that covered other drivers were not included in the analysis as they could not be compared with M-Tool data. Four additional concepts were identified in the interview data that referred to changes in the Nile perch reproduction (one instance), fish migration (two instances), alternative livelihood opportunities (four instances), and transboundary fishing issues (e.g., foreign fishers fishing in Tanzanian waters, six instances).

Each discussed relation between one driver and another or the Nile perch was coded, with the strength of the influence coded when discussions indicated a strength of the connection (Sinval et al., 2020). However, only a few discussions of the relations indicated the strength of the connection, and therefore, the connection strength was omitted from further analysis. When terms were consistently used interchangeably within and across participants (e.g., a participant stated: “Let us say, illegal fishing, the use of destructive fishing gears”), both were coded as the corresponding M-Tool code (i.e., “illegal fishing” became “the use of destructive fishing gear”) It needs to be acknowledged that the assumption that the two terms carry the same meaning may not be true for all participants, which would result in an underestimation of the number of concepts in the mental models of participants. The coding processes resulted in a list of connections between one of the 15 drivers and another driver, or the Nile perch, which could be organized in an edge list similar to the M-Tool mental models.

The coding of the transcript proved to be a challenging task because of imperfect translations from Kiswahili into English and because participants did not tend to express themselves very clearly, leaving room for interpretation. Such challenges are common for multi-language qualitative research (Larkin et al., 2007) and underline the need for a more standardized mental model elicitation tool to avoid such issues. Inter-rater reliability between two independent researchers was assessed, one relying on both the English and the Kiswahili transcripts and one researcher only coding the English transcript. The coders used the definitions for the drivers that were provided to participants in M-Tool (Supplementary Material B). First, the researchers coded 10 randomly selected interviews together to fine-tune the coding system and develop an approach to deal with ambiguous statements. The remaining 20 interviews were subjected to an interrater reliability analysis resulting in an inter-rater agreement of 72.70% [95% CI: (0.66:0.80)]. Although this is below conventional cut-off values of 80%, the agreement could be considered reasonable considering the aforementioned challenges in coding the data. The two coders went through all disagreements, discussed the rationale for their coding approach and agreed on the most appropriate code to resolve each disagreement.

To assess if M-Tool elicited more complex mental models than interviews (Hypothesis 1), we compared the number of drivers that were included in the mental models and the number of connections included per driver. To test the differences in the complexity of the models, we conducted a Poisson model with random subject effects and a binary variable for method type as fixed effects. Deviance residuals were used for model checking and showed no extreme values. To assess the agreement between the composition of M-Tool mental models and interview mental models, we calculate the proportion of drivers and connections that were included in both models, and corresponding confidence intervals.

### Results

Participants tended to be positive about their interaction with M-Tool (“*It felt good to learn how to interact with the tablet*”) and found the instructions clear (“*Instructions were clear, happy to use it*”). Furthermore, they tended to report that they initially found the interaction with the tablet challenging due to their lack of experience with modern technology, but managed to represent their views with M-Tool with the help of the instructions (“*I have never gone to school, and I have never used a big phone [tablet], but even me, I understand how to do this because the instructions are clear and it’s easy*”). The participants reported that the task helped them to think about the issue in more depth and become more aware of the complexity of the issue (“*It made me aware of the many things that influence the Nile perch*”) and possible solutions (“*It gives an awareness on how to stop illegal fishing*”).

Results showed more drivers in M-Tool mental models (*M* = 8.87, SD = 3.72) compared to the interview mental models (*M* = 3.70, SD = 1.39). The likelihood ratio test showed that method type had a significant effect on the number of drivers included in the mental models (likelihood ratio *χ*^2^ = 65.65, *p* < 0.001). M-Tool mental models tended to include 2.4 times more drivers compared to interview mental models [95% CI_log-likelihood_: (1.93:3.00)]. The same analysis was conducted to compare the number of connections per driver between M-Tool and interview mental models and showed no significant differences across the two methods [*M_M-TOOL_* = 1.38, SD*_M-Tool_* = 0.24. *M*_interview_ = 1.31, SD_interview_ = 0.22; likelihood ratio *χ*^2^ = 1.78, *p* = 0.18, 95% CI_log-likelihood_: (−0.04:0.18)]. Hence, these findings confirm that M-Tool mental models are significantly more complex than interview mental models in terms of the number of drivers, but not in terms of the number of connections per driver, partially confirming the first hypothesis.

The analysis showed that 78% of the drivers [95% CI: (0.70:0.85)] in interview mental models were also included in M-Tool mental models. Furthermore, 55% of the connections [95% CI: (0.47:0.63)] in interview mental models tended to be included in M-Tool mental models. These findings highlight a significant agreement in the content of the two types of mental models, confirming the second hypothesis.

**Figure 2:**
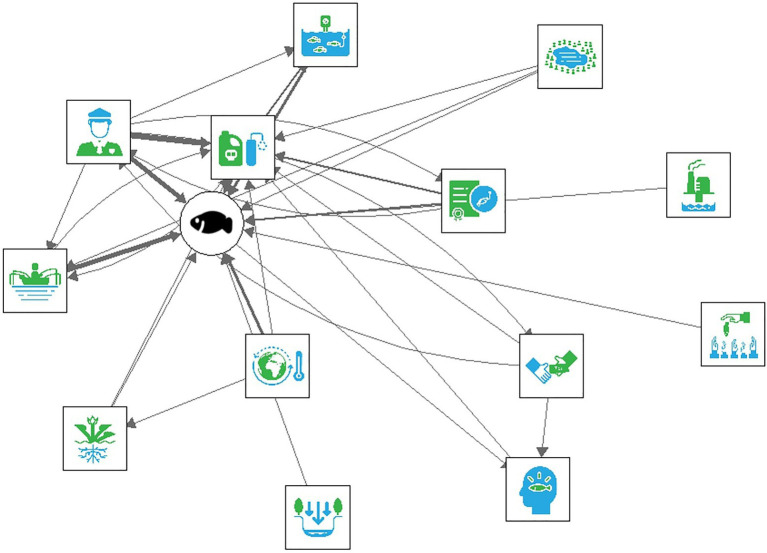
The aggregate interview mental model.

**Figure 3:**
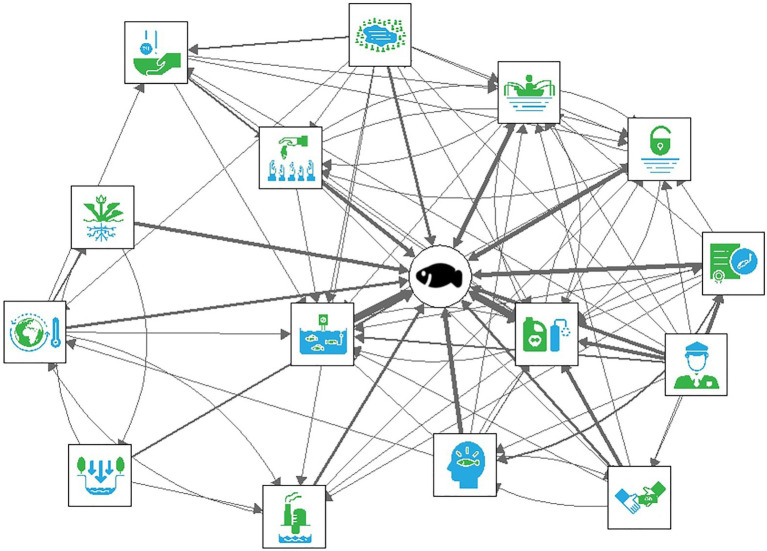
The aggregate M-Tool mental model.

To illustrate these differences in complexity and agreement in composition across the two mental model methods, we have visualized the networks for both types of data. The aggregate interview mental model is displayed in Figure 2, and the aggregate M-Tool mental models is displayed in Figure 3, in which the thickness of the arrows illustrates the sum of all the connections that were included. Figure 3 demonstrates greater connectivity (i.e., more drivers are connected since more drivers were included) compared to Figure 2, but similar drivers tend to be connected across the two models.

## Study 2

### Materials and Methods

Study 2 investigated M-Tool’s construct validity by relating the mental model complexity with participants’ level of education. Since mental model complexity has consistently been found to correlate with participants’ level of education using established mental model methods, education should also correlate with M-Tool mental models complexity if this is a valid method for mental model elicitation. Relating theoretically relevant constructs to validate a new measure is often part of the construct validation process in the domain of personality psychology (Edens and McDermott, 2010; Leary et al., 2013) and has been employed to assess the construct validity of other mental model elicitation methods (Kearney and Kaplan, 1997; Sinval et al., 2020).

#### Participants

For this study, a similar time-sample strategy was employed as for Study 1. Thirteen landing sites were selected at random from a list of all Nile perch landing sites in the Mara and Mwanza region with more than 40 fishing boats targeting Nile perch. The data from Study 1 were not included to ensure the independence of the samples, as the interview process may have affected the mental models produced with M-Tool subsequently. At each landing site, 12–16 fishers were randomly selected from a list of all fishers at the landing site, or from all fishers available at the time of the survey and were financially compensated for their time after completing the study. The sample consisted of 185 fishers, predominantly male (0.5% female), who were experienced fishers (years of fishing_mean_ = 14.68, years of fishing_SD_ = 10.08), functioned as crew members on the fishing boats (63.2%) or were boat-owners (36.8%), and tended to fish with gillnets (59.5%) or longlines/hooks (40.5%). Participants had attained low levels of education (3.8% no education, 78.9% primary education, 16.2% secondary education, 0.5% tertiary/vocational education, 0.5.% university education) and included a wide age range (age_mean_ = 38.64, age_SD_ = 10.73). Similar to Study 1, this is a fairly representative sample for the fishery (Luomba et al., 2013).

#### Procedure

Data were collected directly after the data collection for Study 1, in collaboration with the same local research center and research team. The procedure was identical to Study 1, except for the interview that was omitted for this study. The same demographic items were included in the survey as Study 1.

#### Data Analysis

We analyzed the relation between education and mental model complexity, measured by the number of drivers and the number of connections per driver. Due to the unequal distribution of participants across the five education categories, education was coded into a dummy variable (*M* = 0.17, SD = 0.38), where lower education was coded as 0 (including no education and primary education) and higher education as 1 (including secondary education, tertiary/vocational education, and university education). The number of driver concepts in M-Tool mental models (*M* = 11.03, SD = 2.59), and the number of connections per driver concept in M-Tool mental models (*M* = 1.13, SD = 0.23), served as measures of complexity.

The number of drivers included in the mental model (from maximum 15 possible drivers) was analyzed using a logistic regression model (with grouped binomial data) with education as a predictor. Deviance residuals were used for model checking and showed no extreme values. A probability plot of the number of connections per driver did show deviations from a normal distribution. Therefore, the logarithm of the variable was taken, after which no deviations from normality were detected. The logarithm of the number of connections per driver was analyzed using a linear model with education as a predictor.

### Results

Results showed that the number of drivers in mental models was significantly predicted by education [*β* = 0.30 *p* = 0.01, 95% CI: (0.07:0.54)], see Table 1. Taking the inverse log of this log-odds ratio shows that the odds of including another driver in the mental model was 1.35 times higher among higher educated participants compared to participants who only completed primary school or had no education [95% CI: (1.07:1.71)]. The results showed that education also significantly predicted the number of connections per driver in the mental models (*β* = 0.08, *p* = 0.01). Taking inverse logarithms, this means that the median connections per driver among participants who completed high school or higher was 1.08 times higher than the median connections per driver of participants who only completed primary school or less [95% CI: (1.02:1.15)]. These results confirm that fishers with higher educational backgrounds produced more complex mental models (confirming Hypothesis 3).

**Table 1:** Result of the logistic regression model on the number of drivers and linear model on the number of connections per driver.

	Parameter estimates for the number of drivers				Parameter estimates for the number of connections per driver			
	*β*	*Z*	*p*	95% CI	*β*	*t*	*p*	95% CI
Intercept	0.97	20.81	0.00^***^		0.09	6.70	0.00^***^	
Education	0.30	2.49	0.01^*^	0.07–0.54	0.08	2.57	0.01^*^	0.02–0.14

* *p < 0.05*;

*** *p < 0.001*.

## Discussion

This paper evaluated a new mental model mapping tool, M-Tool, and presents the first step to applying the method with low literacy populations. We compared M-Tool to standard interviewing mental model elicitation methods among rural fishing communities at Lake Victoria, Tanzania, and related M-Tool mental models with participants’ levels of education. The findings suggest that (1) mental models produced with M-Tool tended to be more complex in terms of the number of drivers (but not connections per driver) than mental models obtained through conventional interviewing techniques (partially confirming Hypothesis 1); (2) model composition was similar across the two methods (confirming Hypothesis 2); and (3) participants with higher levels of education tended to produce more complex M-Tool mental models (both in terms of the number of driver and connections per driver) than participants with lower levels of education (confirming Hypothesis 3).

### Validity of M-Tool

The mental model complexity differences across the two methods may be a result of varying levels of cognitive demand required across the two methods. The visual/spatial display created with the visual diagram task in M-Tool is highly compatible with human information processing (Pezdek and Evans, 1979; Suwa and Tversky, 2002). Similarly, learning from knowledge maps has been shown to increase recall of more central ideas, particularly for individuals with low verbal abilities (O’Donnell et al., 2002), and for ill-defined and complex subject-matter content (Tergan, 2005). Moreover, capturing the complexity of mental models may be challenging in interviews because of its high demand on working memory, which has shown to be a restraining factor for complex systems (Hundertmark et al., 2015).

These findings contrast previous research that found no difference in mental model complexity between interview and diagram drawing mental model elicitation methods (Jones et al., 2014). Nevertheless, in the latter study, participants generated the mental model concepts with both methods. Hence, this may suggest that the increased complexity of M-Tool mental models may result from the standardization of the concepts in M-Tool, as participants were provided with the concepts in M-Tool, but not in the interviews. Participants may have felt obliged to use many concepts in their mental model or may have constructed the mental model on the spot rather than reproducing their already existing mental model. Alternatively, the more complex M-Tool mental models may have been a result of the instructions, which were inevitably more extensive for the M-Tool task compared to the interview instructions. Consequently, participants spent more time on the M-Tool task than the interview. The longer time spent on the M-Tool task may have reflected a higher level of engagement with the M-Tool task than the interviews, which may have caused more complex M-Tool mental models than the interview. Furthermore, since the interview was conducted before the M-Tool task, the more comprehensive M-Tool mental model may have also resulted from more cognitive elaboration on the topic. Perhaps the mental models elicited through interview methods represent a mental model with salient features only, while M-Tool may reflect more elaborate thought processes and more detailed perceptions of complex systems. Future research could further investigate whether this is the case by systematically comparing interview and standardized methods, for example, by also providing participants with the mental model concepts in the interview.

The similarity in model composition across the two elicitation methods suggests that the methods tend to measure the same underlying mental model. The findings of this study hence lend support to M-tool’s convergent validity. Despite the fixed set of concepts included in M-Tool, interview mental models tended to focus on similar drivers, suggesting that M-Tool can capture mental models adequately. Participants’ verbal explanations of their cognitive map drawn with M-Tool further validated the representation of the mental models. This finding, together with participants’ positive feedback on their interaction with the tool, suggests that this method is appropriate to elicit mental models of complex systems among participants with lower levels of literacy.

The significant relationship between participants’ education level and mental model complexity provides support for the construct validity of the tool. This finding is in line with previous research that has demonstrated that participants with higher levels of education tend to have more complex mental models of sustainable agriculture (Levy et al., 2018) and climate change (Varela et al., 2020). Furthermore, these findings are also in line with a recent study that showed that informal knowledge acquisition among Lake Victoria stakeholders resulted in different causal beliefs on the Nile perch stock than formal knowledge acquisition (Klein et al., 2021). This is likely because education provides individuals with input for their mental models and the ability to observe or seek out information about systems to further develop their mental model (Greca and Moreira, 2000; Rapp, 2005). Since participants with higher education produced more complex M-Tool mental models in Study 2, this suggests that the tool is able to capture meaningful variations in mental models across individuals.

### Evaluating M-Tool’s Application

The present findings suggest that M-Tool can capture a comprehensive picture of participants’ thinking and the agreement in the structure of the interview and M-Tool mental models support M-Tool’s convergent validity, suggesting it may be a useful tool to capture mental models. Nevertheless, since mental model elicitation methods are likely to influence the resulted representation of the mental model (Payne, 1991), the elicitation method should be carefully selected based on the aim, context, and rationale of the study (Langan-Fox et al., 2000; Moon et al., 2019). Hence, the following sections evaluate the benefits and drawbacks of the tool and discuss when the tool may be most suitable.

#### Benefits

M-Tool may benefit mental model research in three ways. First, due to the use of pictograms and audio instructions, the tool is user-friendly for a wide range of participants. Second, M-Tool standardized format requires little involvement of the interviewer or facilitator, thereby reducing possible noise. Third, there is no need for qualitative interpretations from the researcher with a fixed set of concepts, which is particularly beneficial when assessing populations who may be less able to articulate complex thoughts. This approach facilitates the identification of common ground in mental models as well as differences in the perceptions of complex systems and conservation challenges. M-Tool may therefore be particularly suitable to assess differences across diverse individuals.

#### Limitations

The advantages of a fixed and unified set of concepts also represent a potential disadvantage: they restrict participants with respect to the concepts they can select to create their model. Currently, the tool does not allow participants to add concepts and may limit them from drawing a mental model that accurately reflects their perceptions. Indeed, in Study 1, additional drivers were discussed in the semi-structured interviews, which limited the comparability of the interview mental models with the M-Tool mental models. However, the additional concepts only represented (12/554) ≈ 2% of the coded statements, and model composition across the two methods was similar, suggesting that the set of drivers in M-Tool was fairly representative and exhaustive. To mitigate this limitation of M-Tool, it is crucial that the set of concepts included in the tool is complete and representative of the target population and that participants are asked if they missed any concepts when drawing their cognitive map.

The need to use computer appliances in the M-Tool data collection process may represent another potential challenge, especially with populations with little experience with such devices. Indeed, in the studies presented here, some participants seemed slightly intimidated by the use of a tablet, which required higher levels of engagement of the research assistant than anticipated. However, with research assistants’ scripted help, all except one participant managed to interact with the tablets successfully, and many participants expressed being pleased to have learned how to operate a tablet device. Nevertheless, the web-based version of M-Tool (which does not involve research assistants) may be less suitable for participants with low levels of computer literacy.

Finally, it needs to be acknowledged that there are aspects of the participants’ mental model that M-Tool does not elicit. For example, the tool does not capture the meaning of the connections drawn in the model: the data only reveal which connections the participants envision, and whether these influences are positive or negative if the tool is set up accordingly, but does not specify *how* one concept influences the other. More open mental model elicitation methods, such as interview methods, may be instrumental in uncovering such aspects of mental models. A mixed-methods approach, in which M-Tool quantifies the mental model and interviews produce in-depth insights, may prove particularly fruitful.

### Future Developments

M-Tool has been designed for researchers to compare mental models across diverse participants and is freely available in app stores and on the M-Tool website.^1^ M-Tool can be used to capture perceptions of any system, including the drivers, consequences, actors, actions, and resources. This tool can be instrumental for gaining insights into stakeholders’ perceptions of diverse sustainability challenges, such as those highlighted in the UN sustainable development goals. Particularly, research on sustainability challenges involving many different stakeholders, including those with education and language barriers such as fisheries in this study, may benefit from this tool to map perceptions of those at the heart of these challenges. Such research may produce valuable insights for policymakers to devise effective and well-supported strategies to manage a particular challenge or system.

Future research could use the tool to compare participants’ mental models against an expert model of the system to identify gaps in system understanding (Morgan et al., 2002; Varela et al., 2020). Furthermore, considering that sustainability solutions require the cross-cultural collaborative effort of diverse decision-makers, it is crucial to understand differences in mental models between (groups of) individuals. Differences in mental models may obstruct collaboration (Gurtner et al., 2007; Mathieu et al., 2010), and uncovering such differences in mental models may help identify avenues to overcome barriers and improve collaboration in conservation management (Wood et al., 2012; van den Broek, 2018). Moreover, the tool is suitable to test the within-subject stability of mental models over time. Since mental models are subject to change due (Jones et al., 2011; Pearson and Moon, 2014), assessing the stability of mental models may help separate fluctuations in participants’ understanding of a system over time (e.g., due to external events), from random fluctuations due to unreliable assessment. Alternatively, the tool can be used to assess the impact of an intervention on an individual’s mental model (e.g., a systems-thinking intervention; Goldberg et al., 2020). Such findings will provide more insights into the stability, updating, and nature of mental models.

In sum, we evaluated a standardized and inclusive tool to assess mental models. Two studies supported M-Tool’s convert and construct validity by comparing M-Tool to interviewing methods and by linking M-Tool mental models to education levels. We invite researchers and practitioners to use this tool to investigate mental models of societal or environmental challenges.

## Data Availability Statement

The raw data supporting the conclusions of this article will be made available by the authors, without undue reservation.

## Ethics Statement

Ethical review and approval was not required for the study on human participants in accordance with the local legislation and institutional requirements. The patients/participants provided their written informed consent to participate in this study.

## Author Contributions

KB, JL, and HF contributed to conception and design of the study. KB and JL collected the data. JB performed the statistical analysis. KB wrote the first draft of the manuscript. JL, HF, and JB wrote sections of the manuscript. All authors contributed to the article and approved the submitted version.

## Funding

This work was supported by the Bundesministerium für Bildung und Forschung (grant numbers 01LC1706A and 01LC1822A) and the Field of Focus 4 grant, Heidelberg University (grant number: ZUK 49/Ü 4.1.070).

## Conflict of Interest

The authors declare that the research was conducted in the absence of any commercial or financial relationships that could be construed as a potential conflict of interest.

## Publisher’s Note

All claims expressed in this article are solely those of the authors and do not necessarily represent those of their affiliated organizations, or those of the publisher, the editors and the reviewers. Any product that may be evaluated in this article, or claim that may be made by its manufacturer, is not guaranteed or endorsed by the publisher.

## Footnotes

^1^ www.m-tool.org
