# Supplementary material for: Evaluating the Application of the Mental Model Mapping Tool (M-Tool)
Source: Front Psychol. 2021 Dec 14;12:761882. doi: 10.3389/fpsyg.2021.761882 (PMC8712333; doi:10.3389/fpsyg.2021.761882)
Supplement: Supplementary file 1 [file Table_1.DOCX]

# Appendix B: definition of mental model concepts

**Overpopulation**This images shows overpopulation, this means that are too many people living around Lake Victoria.

**Fishing regulations**This image shows fishing regulations, this means the requirements that the government set for the fishing, such as the size of the boat and the mesh size of the nets.

**Monitoring**This image shows the monitoring, this means the surveillance by the government to check that everyone follows the fishing regulations.

**Open access to the lake**This image shows open access to the lake, this means that anyone can go fishing at Lake Victoria, and that no permits are required.

**Corruption**This image shows corruption, this means that people take bribes

**Poverty**This image shows poverty, this means that people are poor.

**High demand for Nile perch**This image shows a high demand for Nile perch, this means that many people want to buy Nile perch.

**Overfishing**This image shows overfishing, this means that there are too many fishers and boatsat Lake Victoria.

**Use of illegal fishing gear**This image shows the use of illegal fishing gear, this means the use of use of poison, dynamite, nets with small mesh size or small hooks.

**Fishing in breeding grounds**This image shows fishing in breeding grounds, this means fishing at a place where many immature Nile perch are.

**Decreased water level**This images shows a decreased water level, this means that there is less water in Lake Victoria.

**Polluted water**This image show polluted water, this means that the water of Lake Victoria is dirty.”

**Climate change**This image shows climate change, this means that the climate is changing and will continue to change in the future.

**Awareness of sustainable fishing practices**This image shows awareness of sustainable fishing methods, this means people’s that people know how to fish without harming the future fish stock.

**Water hyacinth**This image shows water hyacinth, this is a plant that grows on the lake.
