# Supplementary figures and images for: Evaluating the Application of the Mental Model Mapping Tool (M-Tool)
Source: Front Psychol. 2021 Dec 14;12:761882. doi: 10.3389/fpsyg.2021.761882 (PMC8712333; doi:10.3389/fpsyg.2021.761882)

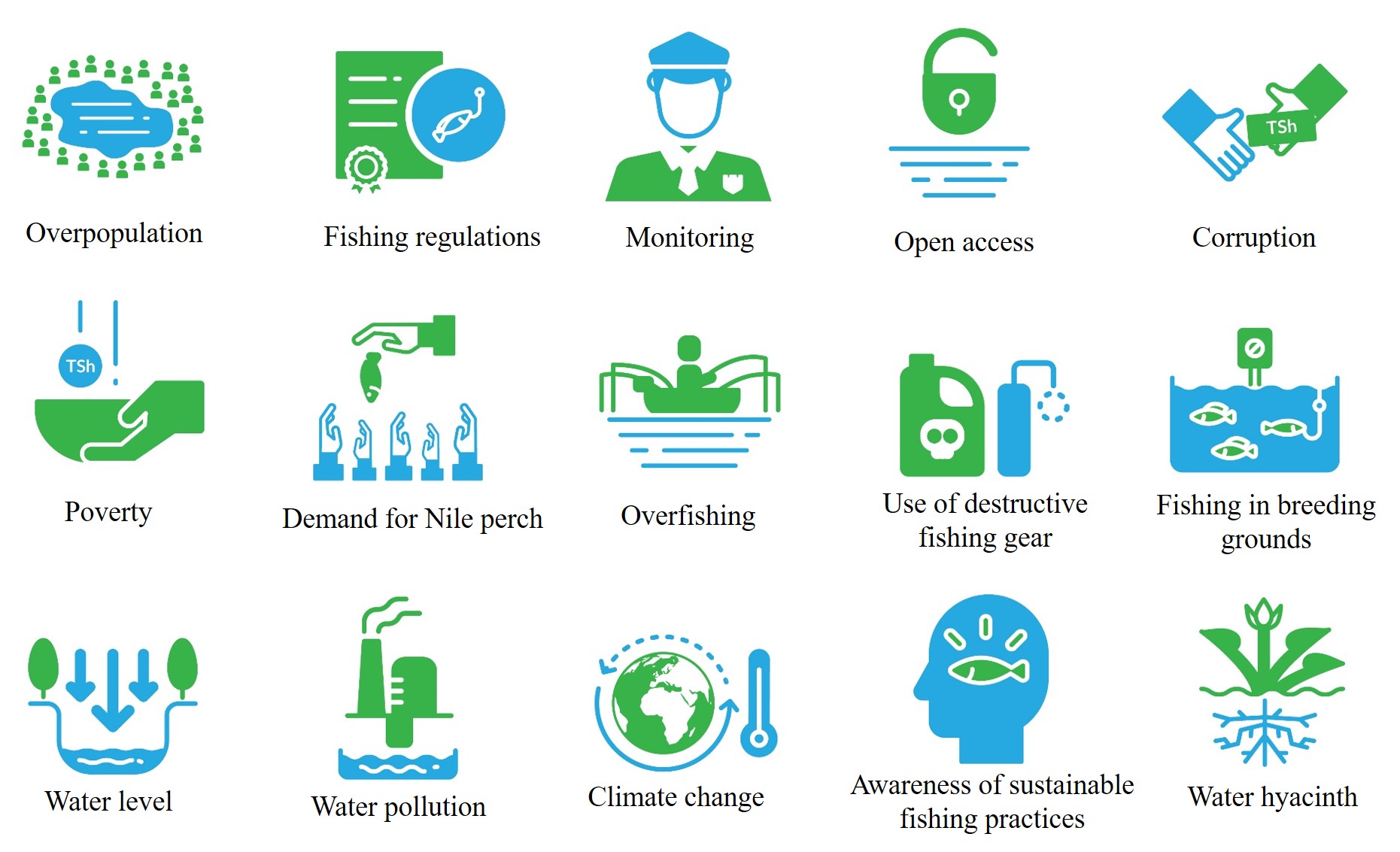

Supplement: Supplementary file 2 [file Image_1.JPEG]
